# Malondialdehyde and Uric Acid as Predictors of Adverse Outcome in Patients with Chronic Heart Failure

**DOI:** 10.1155/2019/9246138

**Published:** 2019-10-09

**Authors:** Ewa Romuk, Celina Wojciechowska, Wojciech Jacheć, Aleksandra Zemła-Woszek, Alina Momot, Marta Buczkowska, Piotr Rozentryt

**Affiliations:** ^1^Department of Biochemistry, School of Medicine with the Division of Dentistry, Medical University of Silesia, Jordana 19 Street, 41-808 Zabrze, Poland; ^2^Second Department of Cardiology, School of Medicine with the Division of Dentistry, Medical University of Silesia, M. C. Skłodowskiej 10 Street, 41-800 Zabrze, Poland; ^3^Institute of Computer Science, Silesian University of Technology, 44-100 Gliwice, Poland; ^4^Department of Toxicology and Health Protection, School of Public Health, Medical University of Silesia, 41-902 Bytom, Poland; ^5^3rd Department of Cardiology, SMDZ in Zabrze, Medical University of Silesia, Silesian Centre for Heart Disease, 41-800 Zabrze, Poland

## Abstract

In chronic heart failure (HF), some parameters of oxidative stress are correlated with disease severity. The aim of this study was to evaluate the importance of oxidative stress biomarkers in prognostic risk stratification (death and combined endpoint: heart transplantation or death). In 774 patients, aged 48-59 years, with chronic HF with reduced ejection fraction (median: 24.0 (20-29)%), parameters such as total antioxidant capacity, total oxidant status, oxidative stress index, and concentration of uric acid (UA), bilirubin, protein sulfhydryl groups (PSH), and malondialdehyde (MDA) were measured. The parameters were assessed as predictive biomarkers of mortality and combined endpoint in a 1-year follow-up. The multivariate Cox regression analysis was adjusted for other important clinical and laboratory prognostic markers. Among all the oxidative stress markers examined in multivariate analysis, only MDA and UA were found to be independent predictors of death and combined endpoint. Higher serum MDA concentration increased the risk of death by 103.0% (HR = 2.103; 95% CI (1.330-3.325)) and of combined endpoint occurrence by 100% (HR = 2.000; 95% CI (1.366-2.928)) per *μ*mol/L. Baseline levels of MDA in the 4^th^ quartile were associated with an increased risk of death with a relative risk (RR) of 3.64 (95% CI (1.917 to 6.926), *p* < 0.001) and RR of 2.71 (95% CI (1.551 to 4.739), *p* < 0.001) for the occurrence of combined endpoint as compared to levels of MDA in the 1^st^ quartile. Higher serum UA concentration increased the risk of death by 2.1% (HR = 1.021; 95% CI (1.005-1.038), *p* < 0.001) and increased combined endpoint occurrence by 1.4% (HR = 1.014; 95% CI (1.005-1.028), *p* < 0.001), for every 10 *μ*mol/L. Baseline levels of UA in the 4^th^ quartile were associated with an increased risk for death with a RR of 3.21 (95% CI (1.734 to 5.931)) and RR of 2.73 (95% CI (1.560 to 4.766)) for the occurrence of combined endpoint as compared to the levels of UA in the 1^st^ quartile. In patients with chronic HF, increased MDA and UA concentrations were independently related to poor prognosis in a 1-year follow-up.

## 1. Introduction

Despite advances in diagnosis and therapy, prognosis in patients with heart failure (HF) is still poor. The prevalence of chronic HF with reduced ejection fraction is increasing, and 1-year mortality is still growing [1]. Evaluation of mortality risk may be a crucial component that can help in choosing patients for invasive treatment (LVAD, HT) [2]. Many parameters are useful in predicting prognosis in patients with chronic HF. Some parameters like natriuretic peptides, age, sex, etiology, NYHA class, left ventricle ejection fraction, and diabetes markedly influence the prognosis of HF [3]. Different biological processes like oxidative stress, inflammation, neurohormonal activation, vascular remodeling, and renal impairment are crucial for the development of the disease [4, 5].

Numerous biomarkers can be a reflection of various pathophysiological pathways [5]. Beyond the well-known N-terminal brain propeptide (NT-proBNP), simple biochemical parameters like natrium, creatinine, or hemoglobin can reflect systemic abnormalities in HF. Several studies have found a correlation between different parameters of oxidative stress and HF severity [6–8]. During oxidative stress, excessive production of reactive oxygen species exceeds the possibility of antioxidant protection. However, their role in the pathogenesis of HF remains unclear [9].

There is still a need to search for a new, easily measured biomarker that can help us understand the HF pathophysiology [5]. Oxidative stress markers, namely, lipid and protein oxidation products, uric acid (UA), and bilirubin, play a multifold role in redox balance during HF, and assessment of these parameters may offer an advantage as compared to other well-known clinical HF markers.

Some studies show serum UA as a poor marker for the prognosis of HF [10, 11]. Impaired oxidative metabolism in HF can be a result of increased xanthine oxidase activity which generates superoxide free radicals proportional to UA synthesis [12–14].

Considering that HF is a state of chronic deterioration of oxidative mechanisms due to enhanced oxidative stress, we assessed if UA, total antioxidant capacity (TAC), total oxidant status (TOS), products of lipid peroxidation (MDA), and protein sulfhydryl groups (PSH), when added to known risk factors, are associated with adverse outcome in patients with stable chronic HF in a 1-year follow-up.

## 2. Study Group and Methods

### 2.1. Patients

In our study, we analyzed the data collected in Prospective Registry of Heart Failure (PR-HF), undertaken since 2003, and Studies Investigating Co-morbidities Aggravating Heart Failure (SICA-HF) described elsewhere [15]. For a prospective cohort study, consecutive patients with primarily chronic systolic HF (LVEF ≤ 40%) were recruited from the patients referred to our Inpatient Clinic as potential candidates for heart transplantation. The primary inclusion criterion was a clinical diagnosis of HF according to published contemporary ESC criteria. Patients received optimal medical pharmacotherapy for at least 3 months before inclusion. Participants were excluded if they had a noncardiac condition resulting in an expected mortality of less than 12 months as judged by the treating physician or had a history of alcohol abuse or known antioxidant supplementation or if they were unable or unwilling to provide informed consent. Study criteria were fulfilled in 1216 PR-HF or SICA-HF. The data of 774 participants (aged 48-59 years) who had completed clinical laboratory assessment were included into final analysis.

### 2.2. Endpoints of the Study

Endpoints of the study were death and combined endpoint (death or urgent heart transplantation).

### 2.3. Consent of the Patient and the Opinion of the Bioethical Commission

All participants provided written informed consent, and the protocol was approved by the participating institution.

### 2.4. Clinical Assessments

At the time of study entry, detailed clinical data were obtained using a standardized questionnaire. Ischemic cardiomyopathy was recognized according to the definition proposed by Felker et al. [16], based on patients who have undergone coronary angiography within six months before inclusion. History of smoking was defined as current or previous use of tobacco products. Comorbidities such as hypertension, hypercholesterolemia, or diabetes mellitus were recognized based on clinical history, current medication, or actual measurements of respective variables. Body mass and height were measured on a day of inclusion visit and body mass indices (BMI) were calculated. The NYHA classification and cardiopulmonary exercise testing CPX were used to assess functional capacity [17]. Two-dimensional transthoracic echocardiography was performed in all patients, and echocardiographic images were acquired in standard views as recommended by the American Society of Echocardiography Committee [18]. Follow-up events including all-cause mortality and cardiac transplantation were prospectively ascertained every 6 months via direct or phone contact with patients or their family members by a dedicated research personnel. In some cases, the exact data of death were obtained from the national identification number database. All participants provided written informed consent, and the protocol was approved by the participating institution.

### 2.5. Biochemical Methods

Venous blood samples obtained at enrollment were processed, separated by centrifugation at 1500 g for 10 minutes, frozen at -70°C, and partially stored at -70°C until time of assay. UA, bilirubin, lipid parameters, blood hemoglobin, and serum iron, sodium, creatinine, glucose, and albumin concentrations were measured by colorimetric method (Roche, Cobas 6000 e 501). NT-proBNP was measured by chemiluminescence method (Roche, Cobas 6000 e 501). Spectrophotometric method by Erel was used to determine total oxidant status (TOS). In this method, the reaction proceeds in an acidic environment and consists of measuring the color intensity of complex of Fe^3+^ ions and Xylenol orange [19]. TOS is expressed in mmol/L.

TAC was measured by colorimetric methods given by Erel, based on 2,2′-azino-bis(3-ethylbenzothiazoline-6-sulfonate) (ABTS+) reaction [20]. In this method, reduced ABTS, a colorless molecule, is oxidized to blue-green ABTS^·^+. After mixing the colored ABTS^·^+ with any substance that can be oxidized, it is reduced to its original colorless reduced form and the reacted substance is oxidized. TAC is expressed in mmol/L.

Oxidative stress index (OSI) is expressed as ratio of TOS to TAC. The OSI was calculated according to the following formula: OSI = [(TOS, mmol/L)/(TAC, mmol/L)] [21]. The concentration of sulfhydryl groups (PSH) in serum was determined by the Koster method using 5,5′-dithiobis (2-nitrobenzoic acid) or DTNB. After reduction by the sulfhydryl group-containing compounds, DTNB gives a yellow-colored anionic 5-thio-2-nitrobenzoic acid [21]. The absorbance was measured with a Shimadzu 1700 UV-VIS spectrophotometer at a wavelength of 412 nm [22]. PSH concentration is expressed in *μ*mol/g protein.

Malondialdehyde (MDA) was measured by Ohkawa's method. The method is based on the reaction of lipid peroxides with thiobarbituric acid with spectrofluorimetric detection. The excitation wavelength was 515 nm, and emission wavelength was 552 nm. MDA concentration was calculated from the standard curve, prepared for 1,1,3,3-tetraethoxypropane, and expressed in *μ*mol/L [23].

### 2.6. Statistical Analysis

The study subjects were divided into groups, for the purpose of the analysis, depending on outcome: A—patients who survived without endpoints, B—patients who died, and C—patients who achieved combined endpoint. Distribution of all continuous variables was evaluated by the Shapiro-Wilk test. The continuous data were presented as median with the first and fourth quartiles (because of abnormal distribution of the data) and were compared using *U* Mann-Whitney test. Categorical data are presented as absolute numbers and percentage and were compared using the chi-square test with Yates correction.

Further estimations of risk were performed using Cox proportional-hazards model. All demographic, clinical, echocardiography, laboratory variables, and medication data were included in a univariate Cox analysis, but only variables with a value of *p* ≤ 0.05 in univariate analysis were included in the multivariate model. The results of the Cox analysis were reported as relative risks with corresponding 95% confidence intervals (CI). Cumulative survival curves were constructed as time to the endpoint by the Kaplan-Meier method, the survival of the groups of patients separated from the quartiles of MDA and UA concentrations was assessed, and the differences were tested for significance by the log-rank test. Differences in the number of achieved endpoints in particular subgroups were also assessed by the Kruskal-Wallis ANOVA test.

Results were considered statistically significant if *p* < 0.05. Lack of statistical significance was indicated by NS (nonsignificant).

Statistical analysis was performed using STATISTICA 13.1 PL (StatSoft, Poland, Cracow).

## 3. Results

### 3.1. Baseline Characteristics of Study Population and Subgroups in Relation to Endpoint

**Figure 1:**
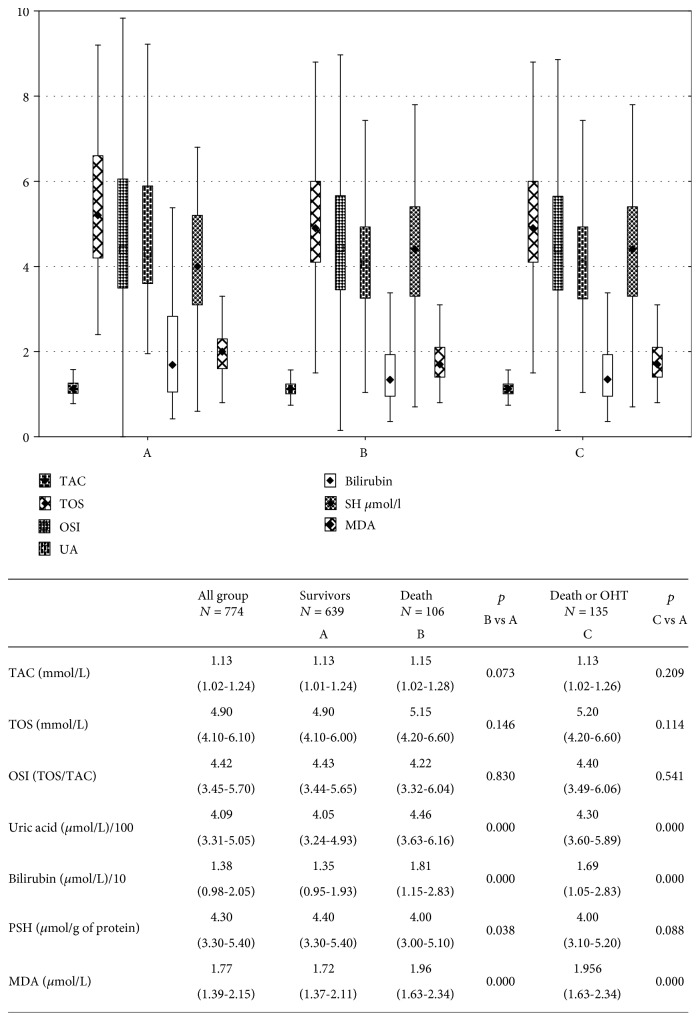
Oxidative stress parameters in examined subgroups depending on prognosis. TAC: total antioxidant capacity; TOS: total oxidant status; OSI: oxidative stress index; MDA: malondialdehyde; PSH: sulfhydryl groups; NS: nonsignificant.

Over a follow-up of one year, there were 106 deaths (group B) and 135 patients reached combined endpoint; there were 29 urgent heart transplantations and 106 deaths (group C). Baseline clinical characteristics for patients enrolled in the study are shown in Table 1 and on Figure 1. The median age was 54 (48-59) years, and 664 patients (85.79%) were male. In general, ischemic HF was more common than nonischemic (61.89% vs. 38.11%) and more frequent in patients who died. The population was classified by the treating cardiologist as having symptomatic systolic HF NYHA class I-II (*n* = 339; 43.8%) and NYHA class III-IV (*n* = 435; 56.2%). The left ventricle was more enlarged in groups B and C with median of ejection fraction in all patients as 24.0% (20.0-29.0). Patients who reached endpoints were more likely to be older (only group B), with lower BMI, longer disease duration, more advanced NYHA class, and lower value of maximum oxygen consumption in cardiopulmonary exercise testing. Diabetes as a comorbid condition was more frequent, and ICD presence was rare. The percentage of use of ACE, loop diuretics, thiazides, statins, and digitalis between groups without endpoint and groups B and C was different while that of beta blockers, angiotensin-2 receptor blockers, mineralocorticoid receptor antagonist, fibrates, and xanthine oxidase inhibitors was similar. In both groups with adverse outcomes, decreased glomerular filtration, natrium and HDL cholesterol concentrations, and significantly increased NT-proBNP concentration were observed. Among the oxidative stress parameters evaluated in our study, UA, bilirubin, and MDA were higher in the groups with unfavorable prognosis. However, TAC, TOS, and OSI were similar for all the groups while PSH was lower in group B.

### 3.2. Association between Redox Parameters and Risk of All-Cause Death

#### 3.2.1. Uni- and Multivariate Cox Regression Analyses

Demographic and clinical parameters, basic laboratory parameters, comorbidities, pharmacotherapy, and oxidative stress-related markers assessed as risk factors for all-cause death in 1-year follow-up in uni- and multivariate Cox regression analyses are presented in Table 2.

In univariate Cox regression analysis, higher levels of TAC (HR = 3.263 per mmol/L; *p* = 0.017), UA (HR = 1.036 per 10 *μ*mol/L; *p* < 0.001), bilirubin (HR = 1.031 per *μ*mol/L; *p* < 0.001), and MDA (HR = 1.967 per *μ*mol/L; *p* < 0.001) and lower levels of PSH (HR = 0.853 per *μ*mol/g protein, *p* = 0.026) were associated with the risk of death. In order to evaluate these oxidative stress parameters in the context of all available clinical information, a final multivariate model was generated that included all significant clinical, laboratory, and treatment predictors. Multivariate analysis revealed that oxidative stress-related risk factors of death were UA (increase of risk (IoR) by 2.1% per 10 *μ*mol/L) and higher serum MDA concentration (IoR by 110.3% per mmol/L). Moreover, in a multivariate analysis, independent risk factors were lower MVO_2_ (IoR by 11.1% per mL/min/kg B.M.), lower LVEF (IoR by 6.27% by every 1%p), lower sodium (IoR by 6.95% per mmol/L), and higher level of NT-proBNP (IoR by 0.9% per 100 pg/mL). Multivariate analysis showed that the following factors are associated with a better prognosis: use of ACE inhibitors (reduction of risk (RoR) by 55.7%) and presence of ICD (RoR by 90.1%).

#### 3.2.2. Kaplan-Meier Survival Analysis

**Figure 2:**
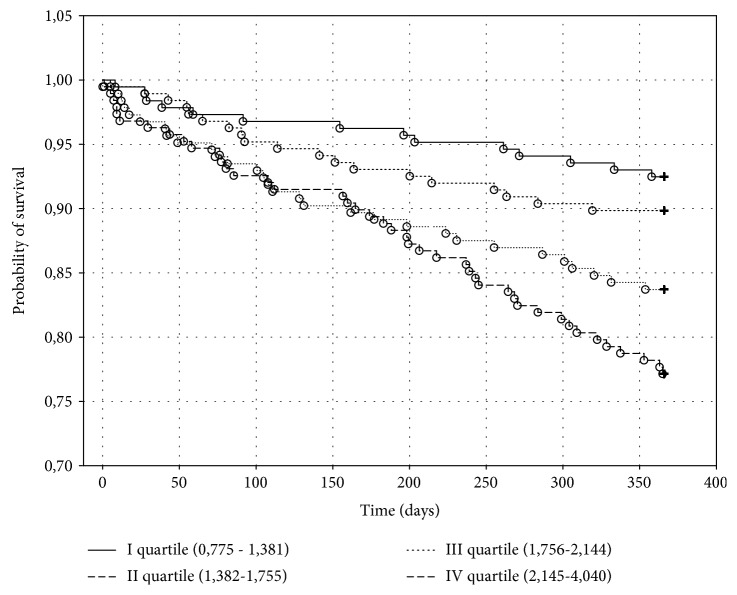
The Kaplan-Meier probability of survival in heart failure patients depending on malondialdehyde concentration in one-year follow-up. Significant coefficient for the whole model: *p* < 0.001.

**Figure 3:**
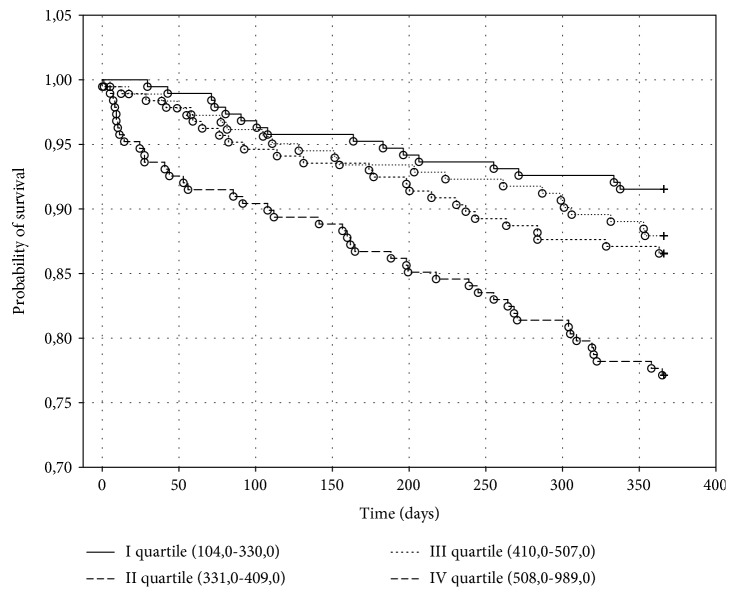
The Kaplan-Meier probability of survival in heart failure patients depending on serum uric acid concentration in one-year follow-up. Significant coefficient for the whole model: *p* < 0.001.

The Kaplan-Meier death curves for biomarker quartiles of MDA and UA are shown, respectively, in Figures 2 and 3.

There were 14 (7.35%) deaths in the first quartile, 19 (10.16%) deaths in the second quartile, 30 (16.30%) deaths in the third quartile, and 43 (22.87%) deaths in the fourth quartile of MDA (*p* < 0.001), whereas there were 16 (8.47%) deaths in the first quartile, 25 (13.44%) deaths in the second quartile, 22 (12.09%) deaths in the third quartile, and 43 (22.87%) deaths in the fourth quartile of UA (*p* < 0.001).

Baseline levels of MDA in the upper quartile were associated with the risk of death with a RR of 3.64 (95% CI (1.917 to 6.926)) compared to levels of MDA in the bottom quartile.

Baseline levels of UA in the upper quartile were associated with the risk of death with a RR of 3.21 (95% CI (1.734 to 5.931)) compared to levels of UA in the bottom quartile.

### 3.3. Association between Redox Parameters and Risk of Combined Endpoint

#### 3.3.1. Uni- and Multivariate Cox Regression Analyses

Risk factors of death or HT in one-year follow-up in univariate and multivariate Cox regression analyses are shown in Table 3. In univariate Cox regression analysis, the following markers reflecting oxidative stress were the risk factors of death or HT: higher concentration of UA (HR = 1.029 per 10 *μ*mol/L; *p* < 0.001), bilirubin (HR = 1.029 per *μ*mol/L; *p* < 0.001), and MDA (HR = 2.037 per *μ*mol/L; *p* < 0.001). Multivariate analysis showed that only two parameters reflecting oxidative stress were independent predictors of combined endpoint, UA (IoR by 1.40% per 10 *μ*mol/L) and higher serum MDA concentration (IoR by 100% per mmol/L). Moreover, in a multivariate analysis, independent risk factors were lower MVO_2_ (IoR by 9.41% per mL/min/kg B.M.), lower LVEF (IoR by 7.35% by every 1%p), lower sodium (IoR by 5.37% per mmol/L), and higher level of NT-proBNP (IoR by 0.7% per 100 pg/mL). Multivariate analysis showed that the following factors are associated with a better prognosis: use of ACE inhibitors (RoR by 43.2%) and presence of ICD (RoR by 82.1%).

#### 3.3.2. Kaplan-Meier Survival Analysis

**Figure 4:**
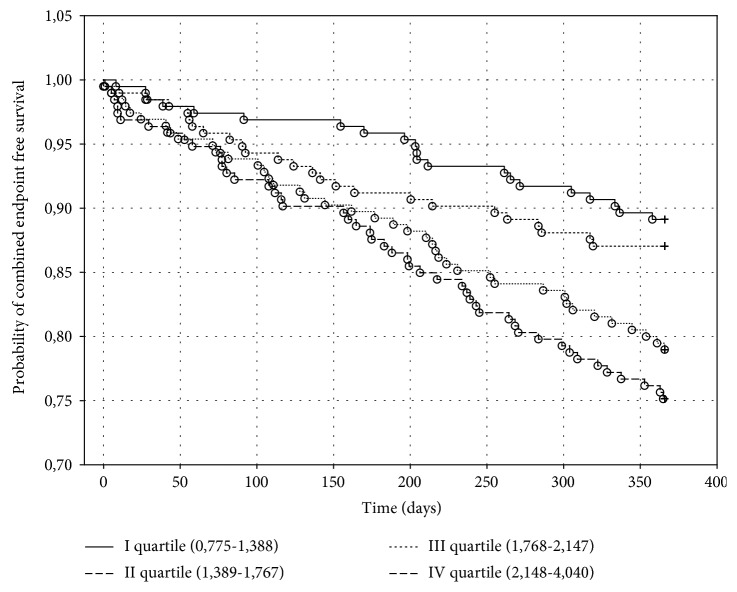
The Kaplan-Meier probability of combined endpoint-free survival in heart failure patients depending on serum malondialdehyde concentration in one-year follow-up. Significant coefficient for the whole model: *p* < 0.001.

**Figure 5:**
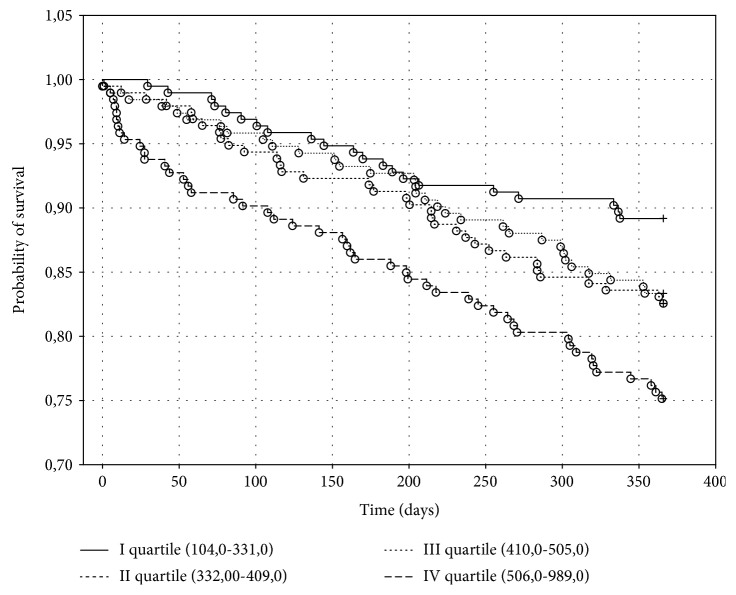
The Kaplan-Meier probability of combined endpoint-free survival in heart failure patients depending on uric acid concentration in one-year follow-up. Significant coefficient for the whole model: *p* < 0.01.

The Kaplan-Meier combined endpoint curves for biomarker quartiles of MDA and UA are shown, respectively, in Figures 4 and 5.

There were 21 (10.88%) deaths or HT in the first quartile, 25 (12.95%) in the second quartile, 41 (21.03%) in the third quartile, and 48 (24.87%) deaths in the fourth quartile of MDA (*p* < 0.001), whereas there were 21 (10.82%) deaths or HT in the first quartile, 34 (17.44%) in the second quartile, 32 (16.679%) in the third quartile, and 48 (24.87%) deaths or HT in the fourth quartile of UA (*p* < 0.001).

Baseline levels of MDA in the 4^th^ quartile were associated with the risk of occurrence of combined endpoint with a RR of 2.71 (95% CI (1.551 to 4.739)) as compared to levels of MDA in the 1^st^ quartile.

Baseline levels of UA in the 4^th^ quartile were associated with the risk of occurrence of combined endpoint with a RR of 2.73 (95% CI (1.560 to 4.766)) as compared to levels of UA in the 1^st^ quartile.

## 4. Discussion

To the best of our knowledge, this is one of the few large prospective studies assessing the effect of oxidative stress parameters including MDA, TAC, TOS, PSH, UA, and bilirubin on prognosis in patients with HF. Increased UA, bilirubin, and MDA were associated with death or OHT in 1-year follow-up. Additionally, higher TAC and lower PSH were risk factors of death. It is worth emphasizing that MDA and UA remained predictors of death and a combined endpoint after considering known demographic, clinical, and biochemical prognostic factors in the analysis.

These data provide a comprehensive evaluation of the prevalence and prognostic importance of redox balance abnormalities in chronic HF due to reduced left ventricular systolic function. Prior studies investigating redox balance abnormalities in HF patients were relatively smaller and evaluated highly selected populations. In several of them, oxidative stress parameter levels were shown to have a significant correlation with hemodynamic measurements, natriuretic serum peptides, and other prognostic parameters of HF [24–26].

There was an inverse relation between the severity of the disease (as measured by the left ventricular ejection fraction, LVEF) and MDA levels, supporting the hypothesis that free radical production is indeed involved in HF and may be linked to its severity [27].

Our results are consistent with those of a study by Radovanovic et al., in which it was reported that high plasma MDA concentration (>8 *μ*mol/L) was a significant independent predictor of mortality in 120 patients with chronic HF in median 13-month follow-up. The patients with MDA above cut-off had eight times higher mortality risk. Protein thiol groups were decreased only in patients with NYHA class IV and were not predictors of death. In our study, PSH concentration was lower in groups of patients who died and was a risk factor in univariate analysis; however, it showed no prognostic significance in multivariate model [28].

Similar predictive significance of MDA levels was also found in other oxidative diseases, such as chronic renal failure [29] and coronary artery disease [30].

The high plasma level of MDA was shown to be a strong independent predictor of cardiovascular disease and mortality in patients on hemodialysis [31].

The most representative subanalysis of the predictive value of MDA in 634 patients from the Prospective Randomized Evaluation of the Vascular Effects of Norvasc Trial showed that MDA is an independent risk factor for a cardiovascular event. Patients with MDA concentration in the highest quartile had a RR of 3.3 for major vascular events (myocardial infarction—fatal, nonfatal, stroke), RR of 4.1 for nonfatal vascular events (unstable angina), and RR of 3.8 for major vascular procedures (percutaneous interventions, coronary artery bypass grafting) as compared to the lowest quartile. In a multivariate analysis, the prognostic value was independent of the other inflammatory biomarkers (IL-6, CRP) and classical risk factors of atherosclerosis [30].

In the present study, patients with MDA levels in the highest quartile had a RR of 3.64 for death and a RR of 2.71 for combined endpoint as compared to the lowest quartile, and MDA was shown to be a strong independent predictor of adverse outcome in HF patients. MDA remained a strong poor prognosis biomarker even after adjustment for other important clinical and laboratory prognostic markers.

Similarly, elevated level of UA was an independent risk factor of death or urgent heart transplantation in 1-year follow-up. Participants with a UA level in the upper quartile had a RR of 3.21 for all-cause death and a RR of 2.73 for combined endpoint as compared to the lowest quartile. Results of UA testing as a predictor of death due to cardiovascular causes and all-cause death in different patient populations are not consistent. In the large epidemiological studies, the adjustment of UA to cardiovascular disease risk factors did not confirm the prognostic value of UA [32]. However, Ioachimescu et al. showed that an increase in serum UA by 1 mg% was associated with 39% increase in the risk of death in univariate Cox regression analysis and 26% increase in the risk of death in multivariate model (after adjusting for age, sex, alcohol consumption, smoking status, body mass index, blood pressure, history of cardiovascular disease, estimated glomerular filtration rate, levels of cholesterol fractions, plasma glucose levels, and other cardiovascular risk factors) in the cohort of patients with high risk of cardiovascular disease [33].

Similarly, in heart failure population, elevated serum UA levels were independently associated with an increased risk of adverse clinical outcomes, both in patients with acute HF [34, 35] and in those with chronic HF [36].

The role of UA as a prognostic factor was confirmed in a study by Iliesiu et al. UA was the most powerful predictor of survival for patients with severe HF (NYHA classes III and IV), with a RR of 7.4 for death in patients with elevated UA (>9.5 mg/dL). Additionally, UA and MDA concentrations were higher in HF patients with lower EF and increased LV filling pressures, and a strong positive correlation between concentrations of MDA and UA was detected [37].

Similarly, the analysis by Wu et al. demonstrated that elevated serum UA levels are an independent predictor of mortality in patients with severe systolic HF [38]. Jankowska et al. documented that hyperuricemia was an independent predictor of death in 18-month follow-up in patients with mild and moderate HF (NYHA I-III) [39]. In a study by Anker et al., it was shown that high serum UA levels were a strong, independent marker of impaired prognosis in patients with moderate to severe HF in derivation and validation group. UA concentration ≥ 565 *μ*mol/L was a valid prognostic marker and useful for metabolic, hemodynamic, and functional staging in chronic HF [10].

Hyperuricemia in HF may be due to the upregulation of xanthine oxidase (XO), a key enzyme in the generation of oxygen free radicals and can reflect an impairment of oxidative metabolism [6]. Significant factors that may also increase the UA concentration in HF are reduced glomerular filtration rate and diuretic use. However, in the present study as well as other previous studies, a significant effect of hyperuricemia on outcomes was observed even after the adjustment for risk factors including renal dysfunction and treatment with thiazide and loop diuretics [40, 41]. Although the question of whether UA is a marker of oxidative stress or a causal factor in the pathogenesis of HF remains unresolved, UA has the potential to serve as an important risk biomarker in heart failure patients. Gotsman et al. demonstrated that increased mortality in patients with UA in upper quartiles (>7.7 mg/dL) and treatment with allopurinol was associated with improved survival [42]. However, in a study by Wu et al., the adjusted risk of death associated with allopurinol use was similar to or slightly worse than the adjusted risk associated with the highest quartile UA among patients without treatment with allopurinol [38]. Givertz et al. showed that xanthine oxidase inhibition in the patients with elevated UA levels and reduced ejection fraction was not effective in improving quality of life, exercise capacity, clinical status, or left ventricular ejection fraction at 24-week follow-up [43]. The results obtained confirm the prognostic properties of UA in HF patients; however, like the study by Givertz et al., we did not show a protective effect of allopurinol therapy.

Serum bilirubin has more recently been described as an independent indicator of right ventricular dysfunction [44]. Allen et al. showed that elevated total bilirubin was the predictor of adverse outcome of cardiovascular death or HF hospitalization and all-cause mortality even after adjustment for other clinical prognostic variables [45]. In a small study by Charniot et al., the authors observed decreased TAC in acute HF patients and suggest that it may be responsible for arrhythmias and complications of AHF [46].

In our study, bilirubin and TAC had strong predictive power in univariate analysis. However, when incorporated into other well-established HF markers, there was no influence of bilirubin and TAC on adverse outcome in HF patients.

## 5. Conclusion

Our study, conducted on a large group of patients, had sufficient numbers of events to investigate a wide range of prognostically important clinical and laboratory variables, especially oxidative markers. Other authors had often assessed the effect of a single oxidative stress marker on the survival of HF patients. We have evaluated a wide range of oxidative stress markers and their impact on mortality and morbidity in HF patients. Finally, malondialdehyde and UA were strongly associated with worse prognosis in this group of patients, even after adjusting for a wide array of other predictors, even well-known NT-proBNP. Proposed biomarkers may be useful in terms of 1-year all-cause mortality. MDA and especially UA tests are widely available, noninvasive laboratory tests. In the light of the results obtained in this study, it seems that validation of elevated MDA and UA levels as independent predictors of outcome has a potentially significant value for risk stratification of chronic HF patients. However, the clinical usefulness of the above findings requires confirmation in subsequent studies.

## Figures and Tables

**Table 1:** Baseline characteristic of examined group and comparison of subgroups separated on the basis of prognosis.

	All groups *N* = 774	Survivors *N* = 639 A	Death *N* = 106 B	*p* B vs. A	Death or OHT *N* = 135 C	*p* C vs. A
General characteristics						
Male *n* (%)	664 (85.79)	549 (85.78)	93 (87.74)	0.726	115 (85.19)	0.932
Age (years)	54.00	54.00	56.00	0.019	55.00	0.191
	48.00-59.00	48.00-59.00	50.00-61.00		50.00-60.00	
BMI (kg/m^2^)	26.24	26.40	25.13	0.035	24.77	0.002
	23.49-29.10	23.81-29.39	21.78-28.89		21.78-28.44	
Duration of symptoms before inclusion (months)	33.85	33.17	51.05	0.002	44.80	0.013
	13.43-69.43	12.93-65.40	18.90-81.00		15.60-79.93	
NYHA class	3.00	2.00	3.00	0.000	3.00	0.000
	2.00-3.00	2.00-3.00	3.00-400		3.00-3.00	
Maximum measured VO_2_ (mL/min/kg B.M.)	15.00	15.50	12.05	0.000	12.35	0.000
	12.20-18.45	12.90-19.10	9.60-14.20		9.70-14.20	
LVEDD (mm)	69.00	69.00	70.00	0.201	72.00	0.046
	64.00-76.00	63.00-76.00	65.00-77.00		65.00-77.00	
LVEDV (mL)	225.0	218.0	250.0	0.002	250.0	0.000
	170.0-285.0	163.0-278.0	198.0-319.50		200.0-320.0	
LVEF (1%)	24.00	25.00	20.00	0.000	20.00	0.000
	20.00-29.00	20.00-30.00	17.00-25.00		17.00-25.00	
Basic biochemistry						
Hemoglobin (g/100 mL)	14.02	14.02	13.94	0.428	14.02	0.788
	13.05-14.99	13.05-14.99	12.73-14.99		12.89-15.15	
Sodium (mmol/L)	136.00	137.00	135.00	0.000	135.00	0.000
	134.00-139.00	134.00-139.00	131.0-137.0		132.0-138.0	
Iron concentration (*μ*mol/L)	17.20	17.50	15.90	0.071	15.11	0.004
	12.00-22.20	12.30-22.50	11.07-20.90		10.80-20.90	
Creatinine clearance (mL/min)	95.09	98.89	75.66	0.000	79.84	0.000
	69.76-119.38	73.53-121.70	59.83-100.12		59.83-104.23	
Serum protein (g/L)	71.00	71.00	72.00	0.105	72.00	0.207
	67.00-75.00	67.00-75.00	67.00-77.00		67.00-77.00	
Albumin (g/L)	42.00	42.00	41.00	0.131	40.00	0.011
	39.00-44.00	39.00-44.00	38.00-44.00		38.00-44.00	
Fasting glucose (mmol/L)	5.50	5.50	5.60	0.305	5.60	0.643
	5.00-6.30	5.00-6.30	5.00-6.90		5.00-6.70	
Total cholesterol (mmol/L)	4.29	4.30	4.27	0.546	4.25	0.431
	3.65-5.21	3.65-5.21	3.57-5.25		3.57-5.19	
Triglycerides (mmol/L)	1.21	1.24	1.12	0.054	1.13	0.122
	0.89-1.73	0.90-1.77	0.84-1.58		0.86-1.60	
Cholesterol HDL (mmol/L)	1.15	1.16	1.09	0.022	1.10	0.018
	0.94-1.41	0.96-1.42	0.83-1.37		0.83-1.37	
Cholesterol LDL (mmol/L)	2.44	2.43	2.51	0.493	2.50	0.578
	1.90-3.16	1.89-3.11	1.92-3.27		1.90-3.26	
NT-proBNP (pg/mL)/100	13.91	12.24	29.64	0.000	30.63	0.000
	6.39-31.81	5.56-25.66	16.20-52.04		15.53-52.65	
Comorbidities						
Ischemic heart disease *n* (%)	479 (61.89)	391 (61.00)	74 (70.00)	0.015	88 (65.00)	0.085
Diabetes *n* (%)	218 (28.17)	169 (26.41)	39 (36.79)	0.037	49 (36.30)	0.027
Arterial hypertension *n* (%)	423 (54.65)	357 (55.78)	55 (51.89)	0.168	66 (48.89)	0.361
Permanent atrial fibrillation *n* (%)	183 (23.64)	146 (22.81)	33 (31.13)	0.084	37 (27.41)	0.307
ICD presence *n* (%)	215 (27.78)	209 (32.66)	4 (3.77)	0.000	6 (4.44)	0.000
Smoker *n* (%)	265 (34.24)	224 (35.00)	33 (31.13)	0.499	41 (30.37)	0.346
Pharmacotherapy						
Beta-blockers *n* (%)	759 (98.06)	629 (98.28)	102 (96.23)	0.244	130 (96.30)	0.196
ACE inhibitors (yes/no)	666 (86.05)	564 (88.13)	77 (72.64)	0.000	102 (75.56)	0.000
ARB (yes/no)	82 (10.59)	69 (10.78)	8 (7.55)	0.372	13 (9.63)	0.762
Loop diuretics (yes/no)	679 (87.73)	549 (85.78)	103 (97.17)	0.002	130 (96.30)	0.001
Thiazide diuretics (yes/no)	99 (12.79)	69 (10.78)	25 (23.58)	0.000	30 (22.22)	0.000
MRA (yes/no)	713 (92.12)	589 (92.03)	96 (90.57)	0.711	124 (91.85)	0.961
Statins (yes/no)	506 (65.37)	428 (66.88)	58 (54.72)	0.019	78 (57.78)	0.052
Fibrates (yes/no)	28 (3.62)	25 (3.91)	2 (1.89)	0.452	3 (2.22)	0.483
Digitalis (yes/no)	352 (45.48)	280 (43.75)	55 (51.89)	0.000	72 (53.33)	0.000
XO inhibitors (yes/no)	285 (36.82)	229 (35.78)	41 (38.68)	0.649	56 (41.48)	0.164

BMI: body mass index; NYHA: New York Heart Association functional class; VO_2_: rate of oxygen consumption; VO_2_ max.: maximum rate of oxygen consumption; VCO_2_: rate of carbon dioxide output; LVEDD: left ventricle end-diastolic diameter; LVEDV: left ventricle end-diastolic volume; LVEF: left ventricle ejection fraction; NT-proBNP: N-terminal pro-B-type natriuretic peptide; CM: cardiomyopathy; ICD: implantable cardioverter defibrillator; ACE inhibitor: angiotensin-converting enzyme inhibitor; ARB: angiotensin-2 receptor blockers; MRA: mineralocorticoid receptor antagonists; XO: xanthine oxidase; NS: nonsignificant.

**Table 2:** Clinical and laboratory parameters as risk factors of death of patients with CHF in one-year follow-up. Uni- and multivariate Cox regression analyses.

	Univariate Cox regression analysis			Multivariate Cox regression analysis		
	HR	95% CI	*p*	HR	95% CI	*p*
General characteristics						
Male (yes/no)	0.993	0.575-1.715	0.981			
Age (1 year)	1.021	1.001-1.041	0.035	1.012	0.984-1.040	0.406
BMI (1 kg/m^2^)	0.957	0.915-1.001	0.053			
Duration of symptoms (1 month)	1.005	1.001-1.008	0.006	1.001	0.996-1.006	0.700
NYHA class (by 1)	3.080	2.334-4.065	0.000	1.023	0.659-1.587	0.921
VO_2_ max. (1 mL/min/kg B.M.)	1.208	1.144-1.247	0.000	1.111	1.035-1.192	0.004
LVEDD (1 mm)	1.009	0.989-1.029	0.400			
LVEDV (1 mL)	1.003	1.001-1.006	0.004			
Lower LVEF (1%)	1.078	1.026-1.111	0.000	1.063	1.017-1.110	0.006
Basic biochemistry						
Hemoglobin **↓** (1 g/100 mL)	1.042	0.927-1.171	0.491			
Sodium **↓** (1 mmol/L)	1.122	1.078-1.168	0.000	1.070	1.012-1.131	0.018
Iron concentration **↓** (1 *μ*mol/L)	1.019	0.994-1.045	0.129			
Creatinine clearance ↓ (1 mL/min)	1.014	1.008-1.020	0.000	1.002	0.993-1.010	0.735
Serum protein (1 g/L)	1.028	0.997-1.060	0.074			
Albumin **↓** (1 g/L)	1.056	1.008-1.105	0.021	1.018	0.953-1.088	0.592
Fasting glucose (1 mmol/L)	1.082	0.988-1.185	0.090			
Total cholesterol (1 mmol/L)	0.959	0.817-1.126	0.609			
Triglycerides (1 mmol/L)	0.831	0.643-1.073	0.156			
Cholesterol HDL (1 mmol/L)	0.608	0.368-1.005	0.053			
Cholesterol LDL (1 mmol/L)	1.079	0.895-1.301	0.425			
NT-proBNP (100 pg/mL)	1.017	1.013-1.021	0.000	1.009	1.000-1.017	0.042
Comorbidities						
Ischemic CM (yes/no)	1.279	0.853-1.919	0.233			
Diabetes (yes/no)	1.531	1.031-2.272	0.035	1.065	0.643-1.764	0.806
Arterial hypertension (yes/no)	0.863	0.590-1.264	0.450			
Atrial fibrillation (yes/no)	1.488	0.986-2.245	0.058			
ICD presence (yes/no)	0.090	0.033-0.244	0.000	0.099	0.031-0.319	0.000
Smoker (yes/no)	0.835	0.554-1.260	0.391			
Pharmacotherapy						
Beta-blockers (yes/no)	0.434	0.160-1.178	0.101			
ACE inhibitors (yes/no)	0.375	0.245-0.575	0.000	0.443	0.257-0.764	0.003
ARB (yes/no)	0.874	0.455-1.675	0.684			
Loop diuretics (yes/no)	5.308	1.684-16.726	0.004	1.759	0.403-7.677	0.452
Thiazide diuretics (yes/no)	2.367	1.512-3.707	0.000	1.757	0.956-3.226	0.069
MRA (yes/no)	0.852	0.445-1.633	0.630			
Statins (yes/no)	0.616	0.420-0.902	0.013	1.082	0.648-1.807	0.763
Fibrates (yes/no)	0.496	0.122-2.010	0.326			
Digitalis (yes/no)	1.370	0.936-2.005	0.105			
XO inhibitors (yes/no)	0.991	0.930-1.056	0.781			
Oxidative stress markers						
TAC (1 mmol/L)	3.263	1.237-8.607	0.017	0.618	0.150-2.546	0.505
TOS (1 mmol/L)	1.052	0.990-1.118	0.102			
OSI (TOS/TAC)	1.018	0.964-1.075	0.516			
Uric acid (10 *μ*mol/L)	1.036	1.024-1.048	0.000	1.021	1.005-1.038	0.012
Bilirubin (1 *μ*mol/L)	1.031	1.020-1.043	0.000	0.991	0.971-1.010	0.339
PSH (1 *μ*mol/g of protein)	0.853	0.741-0.982	0.026	1.097	0.919-1.310	0.306
MDA (1 *μ*mol/L)	1.967	1.410-2.742	0.000	2.103	1.330-3.325	0.001

BMI: body mass index; NYHA: New York Heart Association functional class; VO_2_ max.: maximum rate of oxygen consumption; LVEDD: left ventricle end-diastolic diameter; LVEDV: left ventricle end-diastolic volume; LVEF: left ventricle ejection fraction; NT-proBNP: N-terminal pro-B-type natriuretic peptide; CHF: chronic heart failure; ICD: implantable cardioverter defibrillator; ACE inhibitor: angiotensin-converting enzyme inhibitor; ARB: angiotensin-2 receptor blockers; MRA: mineralocorticoid receptor antagonists; XO: xanthine oxidase; TAC: total antioxidant capacity; TOS: total oxidant status; OSI: oxidative stress index; MDA: malondialdehyde; PSH: sulfhydryl groups; NS: nonsignificant.

**Table 3:** Clinical and laboratory parameters as risk factors of death or OHT of patients with CHF in one-year follow-up. Uni- and multivariate Cox regression analyses.

	Univariate Cox regression analysis			Multivariate Cox regression analysis		
	HR	95% CI	*p*	HR	95% CI	*p*
General characteristics						
Male (yes/no)	1.003	0.617-1.629	0.992			
Age (1 year)	1.009	0.993-1.026	0.272			
BMI (1 kg/m^2^)	0.946	0.909-0.984	0.006	0.976	0.924-1.030	0.377
Duration of symptoms (1 month)	1.004	1.001-1.007	0.019	1.001	0.997-1.005	0.683
NYHA class (by 1)	2.815	2.201-3.601	0.000	1.122	0.764-1.648	0.556
VO_2_ max. (1 mL/min/kg B.M.)	1.192	1.138-1.248	0.000	1.094	1.032-1.161	0.003
LVEDD (1 mm)	1.014	0.996-1.032	0.139			
LVEDV (1 mL)	1.004	1.002-1.005	0.000			
Lower LVEF (1%)	1.087	1.057-1.119	0.000	1.075	1.025-1.117	0.000
Basic biochemistry						
Hemoglobin **↓** (1 g/100 mL)	1.006	0.907-1.115	0.913			
Sodium **↓** (1 mmol/L)	1.099	1.058-1.142	0.000	1.054	1.003-1.106	0.037
Iron concentration **↓** (1 *μ*mol/L)	1.028	1.004-1.050	0.021	1.020	0.990-1.052	0.184
Creatinine clearance **↓** (1 mL/min)	1.013	1.007-1.018	0.000	1.000	0.992-1.007	0.912
Serum protein **↓** (1 g/L)	0.980	0.954-1.007	0.152			
Albumin **↓** (1 g/L)	1.067	1.025-1.111	0.002	1.011	0.923-1.072	0.707
Fasting glucose (1 mmol/L)	1.068	0.983-1.160	0.123			
Total cholesterol (1 mmol/L)	0.965	0.838-1.112	0.624			
Triglycerides (1 mmol/L)	0.855	0.687-1.065	0.163			
Cholesterol HDL (1 mmol/L)	0.594	0.379-0.932	0.023	1.140	0.705-1.843	0.594
Cholesterol LDL (1 mmol/L)	1.079	0.915-1.273	0.365			
NT-proBNP (100 pg/mL)	1.016	1.013-1.020	0.000	1.007	1.000-1.015	0.038
Comorbidities						
Ischemic CM (yes/no)	1.179	0.828-1.681	0.360			
Diabetes (yes/no)	1.490	1.049-2.116	0.026	1.170	0.750-1.825	0.489
Arterial hypertension (yes/no)	0.796	0.568-1.115	0.184			
Atrial fibrillation (yes/no)	1.265	0.867-1.847	0.223			
ICD presence (yes/no)	0.127	0.059-0.271	0.000	0.179	0.082-0.391	0.000
Smoker (yes/no)	0.803	0.557-1.160	0.242			
Pharmacotherapy						
Beta-blockers (yes/no)	0.541	0.200-1.464	0.227			
ACE inhibitors (yes/no)	0.447	0.301-0.665	0.000	0.568	0.345-0.935	0.026
ARB (yes/no)	0.899	0.508-1.593	0.715			
Loop diuretics (yes/no)	4.984	1.843-13.477	0.002	1.832	0.552-6.079	0.323
Thiazide diuretics (yes/no)	2.179	1.452-3.270	0.000	1.579	0.909-2.744	0.105
MRA (yes/no)	1.079	0.567-2.053	0.817			
Statins (yes/no)	0.710	0.504-1.000	0.051			
Fibrates (yes/no)	0.578	0.184-1.814	0.347			
Digitalis (yes/no)	1.469	1.047-2.061	0.026	0.828	0.541-1.266	0.383
XO inhibitors (yes/no)	0.993	0.955-1.034	0.746			
Oxidative stress markers						
TAC (1 mmol/L)	2.405	0.993-5.824	0.052			
TOS (1 mmol/L)	1.044	0.986-1.104	0.139			
OSI (TOS/TAC)	1.016	0.966-1.068	0.537			
Uric acid (10 *μ*mol/L)	1.029	1.018-1.040	0.000	1.014	1.001-1.028	0.042
Bilirubin (1 *μ*mol/L)	1.029	1.018-1.039	0.000	0.995	0.977-1.013	0.580
PSH (1 *μ*mol/g of protein)	0.891	0.786-1.011	0.073			
MDA (1 *μ*mol/L)	2.037	1.517-2.735	0.000	2.000	1.366-2.928	0.000

BMI: body mass index; NYHA: New York Heart Association functional class; VO_2_ max.: maximum rate of oxygen consumption; LVEDD: left ventricle end-diastolic diameter; LVEDV: left ventricle end-diastolic volume; LVEF: left ventricle ejection fraction; NT-proBNP: N-terminal pro-B-type natriuretic peptide; CHF: chronic heart failure; ICD: implantable cardioverter defibrillator; ACE inhibitor: angiotensin-converting enzyme inhibitor; ARB: angiotensin-2 receptor blockers; MRA: mineralocorticoid receptor antagonists; XO: xanthine oxidase; TAC: total antioxidant capacity; TOS: total oxidant status; OSI: oxidative stress index; MDA: malondialdehyde; PSH: sulfhydryl groups; NS: nonsignificant.

## Data Availability

The original data is available after contact with the corresponding author.
